# Osmotin attenuates LPS-induced neuroinflammation and memory impairments via the TLR4/NFκB signaling pathway

**DOI:** 10.1038/srep24493

**Published:** 2016-04-20

**Authors:** Haroon Badshah, Tahir Ali, Myeong Ok Kim

**Affiliations:** 1 Division of Applied Life Science (BK 21), College of Natural Sciences (RINS), Gyeongsang National University, Jinju, 660-701, Republic of Korea

## Abstract

Toll-like receptor 4 (TLR4) signaling in the brain mediates autoimmune responses and induces neuroinflammation that results in neurodegenerative diseases, such as Alzheimer’s disease (AD). The plant hormone osmotin inhibited lipopolysaccharide (LPS)-induced TLR4 downstream signaling, including activation of TLR4, CD14, IKKα/β, and NFκB, and the release of inflammatory mediators, such as COX-2, TNF-α, iNOS, and IL-1β. Immunoprecipitation demonstrated colocalization of TLR4 and AdipoR1 receptors in BV2 microglial cells, which suggests that osmotin binds to AdipoR1 and inhibits downstream TLR4 signaling. Furthermore, osmotin treatment reversed LPS-induced behavioral and memory disturbances and attenuated LPS-induced increases in the expression of AD markers, such as Aβ, APP, BACE-1, and p-Tau. Osmotin improved synaptic functionality via enhancing the activity of pre- and post-synaptic markers, like PSD-95, SNAP-25, and syntaxin-1. Osmotin also prevented LPS-induced apoptotic neurodegeneration via inhibition of PARP-1 and caspase-3. Overall, our studies demonstrated that osmotin prevented neuroinflammation-associated memory impairment and neurodegeneration and suggest AdipoR1 as a therapeutic target for the treatment of neuroinflammation and neurological disorders, such as AD.

Neuroinflammation is associated with several neurodegenerative disorders, such as Alzheimer’s disease (AD), Parkinson’s disease, and Huntington’s disease. Lipopolysaccharide (LPS) is an endotoxin from the outer membrane of Gram-negative bacteria that activates the immune system. Systemic administration of LPS evokes inflammatory processes in the body, which cause detrimental effects on the brain and other vital organs. *In vitro* and *in vivo* studies demonstrated that LPS induced inflammation via the up-regulation of different proinflammatory mediators, such as nitric oxide species (NOS), prostaglandin E2 (PGE2), and cyclooxygenase (COX)-2, and proinflammatory cytokines, including interleukin-1 (IL-1), IL-6, and tumor necrosis factor-α (TNF-α)^1^,^2^,^3^.

Toll-like receptors (TLRs) play important roles in the sensing of body pathogens and macrophages and the initiation of immune responses. LPS is primarily recognized by the CD14/TLR-4 receptor complex, which is expressed on microglia and astrocytes in the CNS^1^,^4^,^5^. The downstream signal transduction of this receptor complex activates the TLR4/nuclear factor (NF)-κB pathway, which leads to the generation of inflammatory mediators and the production of neuroinflammation and neurodegeneration^6^,^7^. Neuroinflammation induced by a single intraperitoneal (i.p.) injection of LPS persists for 10 months in mouse brain and produces neurodegenerative effects^8^. The neuroinflammatory cytokines and chemokines that are released as result of LPS administration induce amyloidogenesis via an increase in β-secretase (BACE) activity^9^. Other studies demonstrated a significant increase in the concentration of neuroinflammatory markers, such as proinflammatory cytokines, chemokines, and prostaglandins, in AD brain^10^,^11^. Earlier studies described an excessive production of IL-1, IL-6, TNF-α, macrophage-derived inflammatory factor (MIP)-α, and superoxide free radicals in AD glial cells^12^,^13^. Sambamurti *et al.*^14^ demonstrated that NFκB regulates amyloid beta (Aβ) formation, and the promoter region of BACE-1 contains an NFκB binding site^14^. Several studies confirmed that LPS administration impaired spatial learning performance, increased Aβ production, and disrupted synaptic function^15^,^16^. Akiyama *et al.*^12^ demonstrated that transgenic animals overexpressing IL-6 and TNF-α exhibited cognitive impairment^12^.

Several studies demonstrated that different anti-inflammatory agents attenuated the neuroinflammatory effects of LPS^16^,^17^. However, fewer reports elucidated the exact mechanism of the inhibition of LPS-induced neurotoxicity. Garate *et al.*^6^ recently demonstrated that an inhibitor of TLR4, TAK242, antagonized the neuroinflammatory effects of LPS^6^. Adiponectin administration to cardiomyocytes interferes with TLR4 signaling and protects against LPS-induced cardiac inflammation^18^. Osmotin is a protein extracted from the tobacco plant that is a structural and functional homolog of the mammalian hormone adiponectin^19^,^20^. Therefore, we hypothesized that osmotin would interact with the TLR4/CD14 receptor complex and inhibit the downstream inflammatory processes. This study demonstrated that systemic LPS administration induced neuroinflammation and an AD-like pathology in the hippocampus of adult mice. We previously demonstrated the neuroprotective effect of osmotin against ethanol- and amyloid-beta-induced neurotoxicity^21^,^22^. The present study evaluated the neuroprotective effect of osmotin against LPS-induced neuroinflammation and the possible mechanism of osmotin in the attenuation of the neurotoxic effects of LPS.

## Results

### Osmotin prevents LPS-induced neuroinflammatory processes via inhibition of the TLR4-NFκB pathway

**Figure 1:**
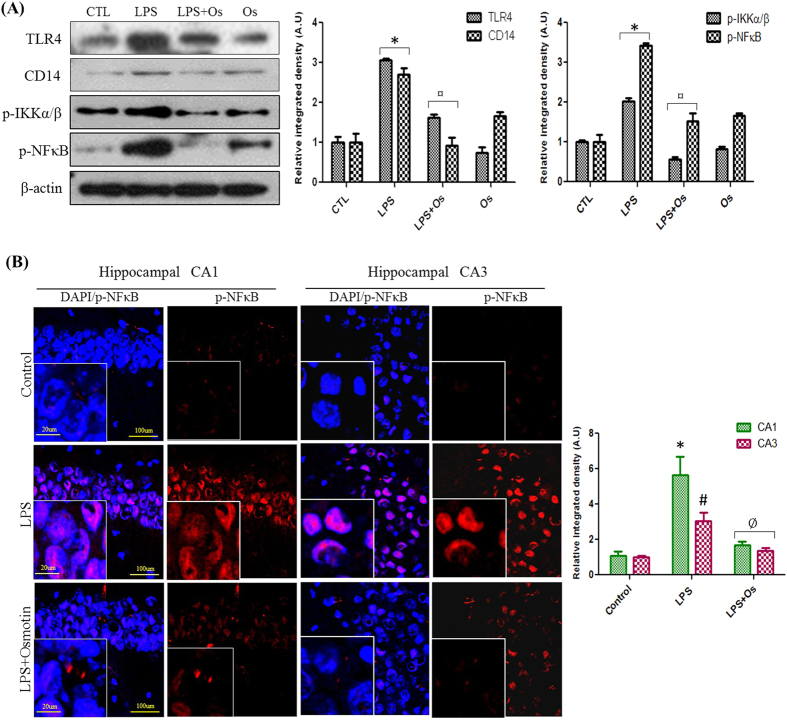
Osmotin prevents LPS-induced neuroinflammatory processes via inhibition of the TLR4-NFκB pathway (**A**) Shown are representative Western blots probed with TLR4, CD14, p-IKKα/β, and p-NFκB antibodies from the hippocampus of adult mice. The density values are expressed in arbitrary units as the mean ± SEM of the indicated proteins (n = 5 animals per group). (**B**) Shown are representative photomicrographs of immunofluorescence analyses of p-NFκB-positive cells in the experimental groups. Images are representative stains obtained in sections prepared from at least 5 animals per group. All of the panels representing the CA1, CA3, and DG regions of the hippocampus show p-NFκB-stained brain tissue at a magnification of 10× objective field (scale bar = 100 μm) and magnification of 40× objective field (scale bar = 20 μm). Symbol representation for the treatment groups and level of significance are described in the data analysis section of the Materials and Methods.

The structure-activity relationship of LPS clearly elucidated the binding of LPS with TLR4 receptors, and the crystal structure of the TLR4-MD-2-LPS complex demonstrated the involvement of TLR4 receptor signaling in the progression of LPS-induced inflammatory activities^23^. CD14 receptors also amplify LPS responses, and TLR4/CD14 complexes serve as LPS signaling receptors^24^. Western blot analysis were performed to evaluate the effect of osmotin on LPS-induced activation of TLR4 and CD14 receptors. Our results demonstrated that LPS injection significantly increased TLR4 and CD14 expression compared to the control group in the hippocampus of adult mice and that osmotin treatment significantly reduced the LPS-induced elevated TLR4 and CD14 expression (Fig. 1A).

Several studies demonstrated that LPS-TLR4 signaling mediated the phosphorylation and nuclear translocation of the transcription factor NFκB^25^. IKKβ phosphorylation is primarily involved in the NFκB activity that is triggered by various proinflammatory stimuli^26^. Western blot and immunofluorescence analyses were performed to explore the anti-inflammatory effect of osmotin on LPS-induced NFκB activation. Western blot analysis revealed that systemic LPS administration significantly increased IKKα/β phosphorylation and the nuclear translocation of NFκB-p65 compared to the vehicle-treated group. Osmotin and LPS administration significantly decreased hippocampal levels of p-IKKα/β and NFκB compared to the LPS-treated group (Fig. 1A). We also demonstrated that osmotin inhibited LPS-induced activation of TLR4 and NFκB in the cortex of adult mice (Fig. S1). NFκB localization after LPS and osmotin treatment was also observed using confocal microscopy. An apparent increase in the CA1, CA3 and DG regions of the hippocampus was observed in LPS-treated mice compared to the control group. Osmotin treatment significantly attenuated LPS-induced NFκB nuclear translocation in the hippocampus compared to the LPS-treated group (Fig. 1B).

### Anti-inflammatory mechanism of osmotin against LPS-induced neuroinflammation in BV2 microglial cells

**Figure 2:**
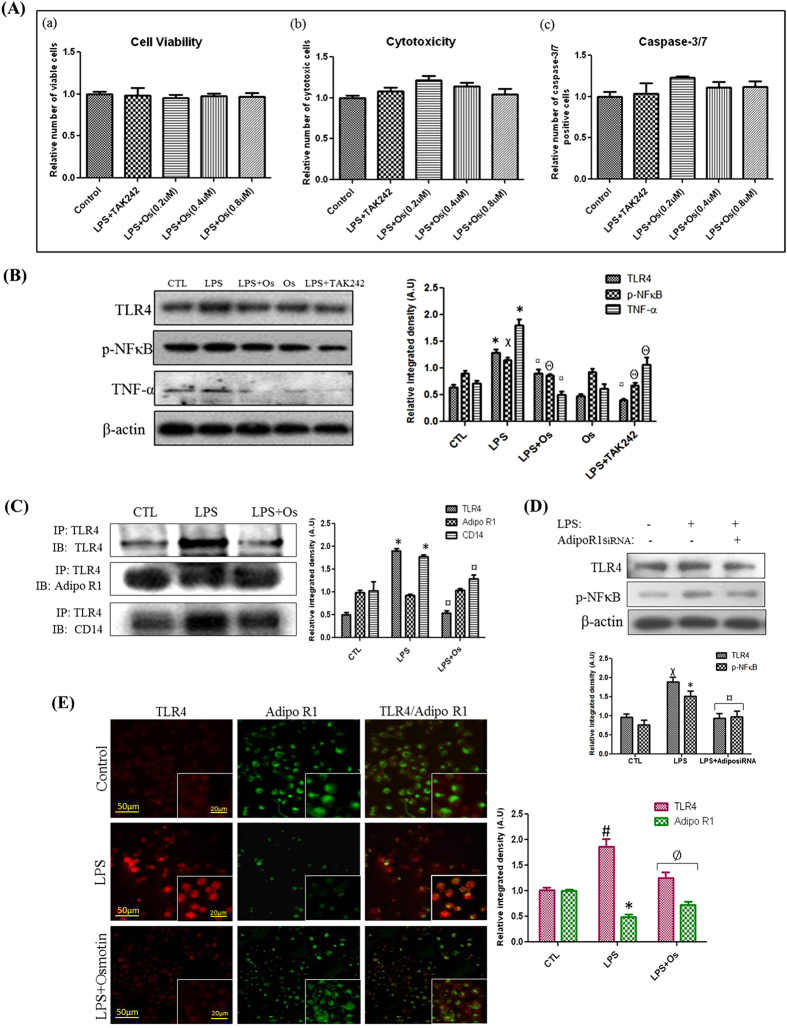
Anti-inflammatory mechanism of osmotin against LPS-induced neuroinflammation in BV2 microglial cells (**A**) Shown are representative histograms for (a) Cell Viability, (b) Cytotoxicity, and (c) Caspase-3/7 assays performed under experimental conditions as mentioned in the Materials and Methods section. (**B**) Shown are representative Western blots probed with TLR4, p-NFκB, and TNF-α antibodies in microglial cells. (**C**) Shown are immunoprecipitation results followed by representative immunoblots as mentioned in the Materials and Methods section. AdipoR1 colocalizes with the TLR4/CD14 signaling complex in microglial cells. (**D**) Shown are representative Western blots probed with TLR4 and p-NFκB antibodies in microglial cells. AdipoR1 siRNA transfection significantly inhibited LPS-induced inflammation. The density values are expressed in arbitrary units as the mean ± SEM for the indicated proteins (n = 5 per group). (**E**) The immunofluorescence images indicate localization of TLR4 (red panels), AdipoR1 (green panels) and merged TLR4 and AdipoR1 (merge panels). The images are representative of staining obtained in sections prepared from at least 5 animals per group (low magnification = 10x, scale bar: low zoom = 50 μm and high zoom = 20 μm). Symbols for treatment groups and levels of significance are mentioned in the data analysis section of the Materials and Methods.

Microglial cells are the primary LPS-responsive cells in the CNS. BV2 microglial cells were used for apoptotic, Western blot, immunoprecipitation, and immunofluorescence assays to elucidate the functional neuroprotective and anti-inflammatory roles of osmotin against LPS-induced neuroinflammation. An Apo-Tox Glo triplex assay kit was used to investigate microglial cell viability, toxicity, and caspase 3/7 activity in control, LPS-treated, osmotin-treated, and LPS and TAK242-treated groups. TAK242 is a well-known inhibitor of the TLR4 receptor, and it inhibits the downstream neuroinflammation following TLR4 activation^13^. Our results demonstrated that osmotin possessed potent neuroprotective activity following LPS treatment via the maintenance of cell viability, reduction of cytotoxicity, and inhibition of caspase 3/7 activation, similarly to LPS and TAK242 treatment. We also demonstrated that 0.4 μM osmotin was the optimum dose for adequate neuroprotective activity of the three doses used (i.e., 0.2 μM, 0.4 μM, and 0.8 μM) (Fig. 2A).

Western blot analysis and immunoprecipitation were performed in microglial cells to investigate the anti-inflammatory mechanisms of osmotin. Osmotin significantly attenuated LPS-induced increases in TLR4, p-NFκB, and TNF-α in microglial cells (Fig. 2B), which supported the anti-inflammatory activity of osmotin. Our immunoprecipitation results demonstrated an interaction of AdipoR1 with TLR4 and its co-receptor CD14 in control, LPS-treated, and LPS plus osmotin-treated microglial cells. Our results demonstrated that LPS treatment enhanced the protein levels of AdipoR1 and the TLR/CD14 complex. We demonstrated that osmotin binding to AdipoR1 inhibited the downstream signaling of TLR4 by dissociating the ligand-receptor complex from the major TLR4/CD14 receptor complex (Fig. 2C). The inhibition of TLR4 downstream signaling was further evaluated using small interfering RNAs (siRNAs) of AdipoR1 treatment to microglial cells, and our results demonstrated that AdipoR1siRNA treatment significantly inhibited the expression of LPS-induced inflammatory markers, such as TLR4 and p-NFκB (Fig. 2D). We also demonstrated an enhanced expression of AdipoR1 in BV2 microglial cells following osmotin and LPS treatment (Fig. S2).

We performed double-labeled immunofluorescence to assess localization and distributional changes of TLR4 and AdipoR1 in microglial cells. Our results revealed the colocalization of TLR4 and AdipoR1 receptors as a clear overlap of fluorescent signals of these receptors in different treatment groups. Immunofluorescence results also demonstrated an increased expression of TLR4 and decreased expression of AdipoR1 in LPS-treated groups compared to the control group. Notably, osmotin reversed the LPS-induced expression trends of TLR4 and AdipoR1 and decreased TLR4 expression and increased AdipoR1 expression compared to the LPS-treated group in microglial cells (Fig. 2E). Overall, these results support the involvement of AdipoR1 in the downstream signal processing of the TLR4/CD14 receptor complex.

### Osmotin inhibits LPS-induced COX-2, iNOS, TNF-α, and IL-1β expression

**Figure 3:**
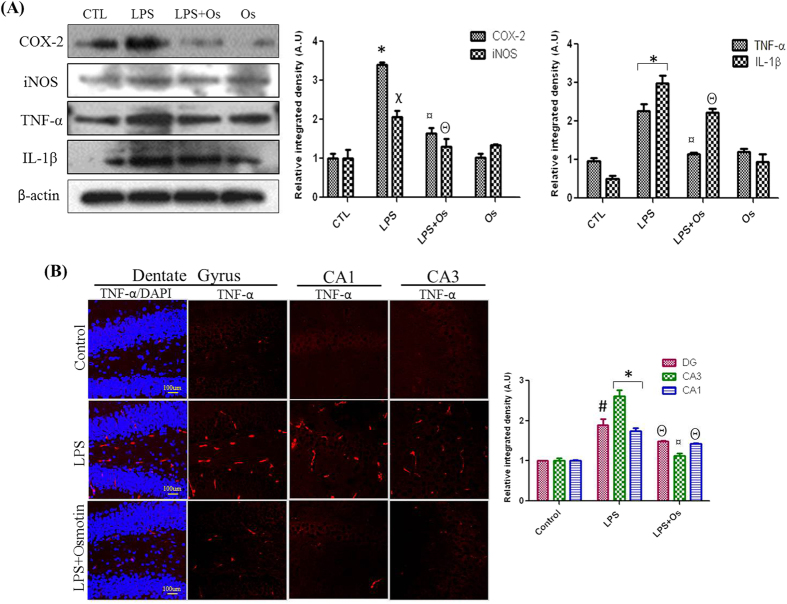
Osmotin inhibits LPS-induced COX-2, iNOS, TNF-α and IL-1β expression (A) Shown are representative western blots probed with antibodies of COX-2, iNOS, TNF-α and IL-1β in the hippocampus of adult mice. The protein bands were quantified using sigma gel software. The density values are expressed in arbitrary units as the mean ± SEM for the indicated proteins (n = 5 animals per group). (B) Showed are representative photomicrographs of immunofluorescence analysis of Tnfα positive cells in the experimental groups. Images are representative of staining obtained in sections prepared from at least 5 animals per group. Panels representing DG, CA1 and CA3 region of hippocampus showed TNF-α stained brain tissue at magnification 10× objective field, scale bar= 100 µm. Symbols for treatment groups and level of significance are mentioned in data analysis section of material methods.

NFκB activation is the primary event in the progression of the proinflammatory signaling pathway. LPS administration induced iNOS activation and the release of proinflammatory mediators, such as TNF-α and IL-1β, via the NFκB–MAPK pathway^27^. Font-Nieves *et al.*^3^ demonstrated that COX-2 activation followed an NFκB-dependent pathway in LPS-treated samples^3^. Hippocampal protein expression levels of COX-2, iNOS, TNF-α, and IL-1β were evaluated to examine the effects of LPS and osmotin on the release of inflammatory mediators. Our results demonstrated that LPS treatment for the indicated period induced a higher level of protein expression compared to the vehicle-treated group, and osmotin administration decreased the protein levels of these inflammatory markers in the hippocampus of LPS-injected mice (Fig. 3A). Immunofluorescence detection of TNF-α revealed that osmotin effectively attenuated LPS-induced increases of TNF-α levels in the DG, CA1, and CA3 regions of the hippocampus (Fig. 3B).

### Effects of osmotin on LPS-induced activation of astrocytes and microglia in the hippocampus of adult mice

**Figure 4:**
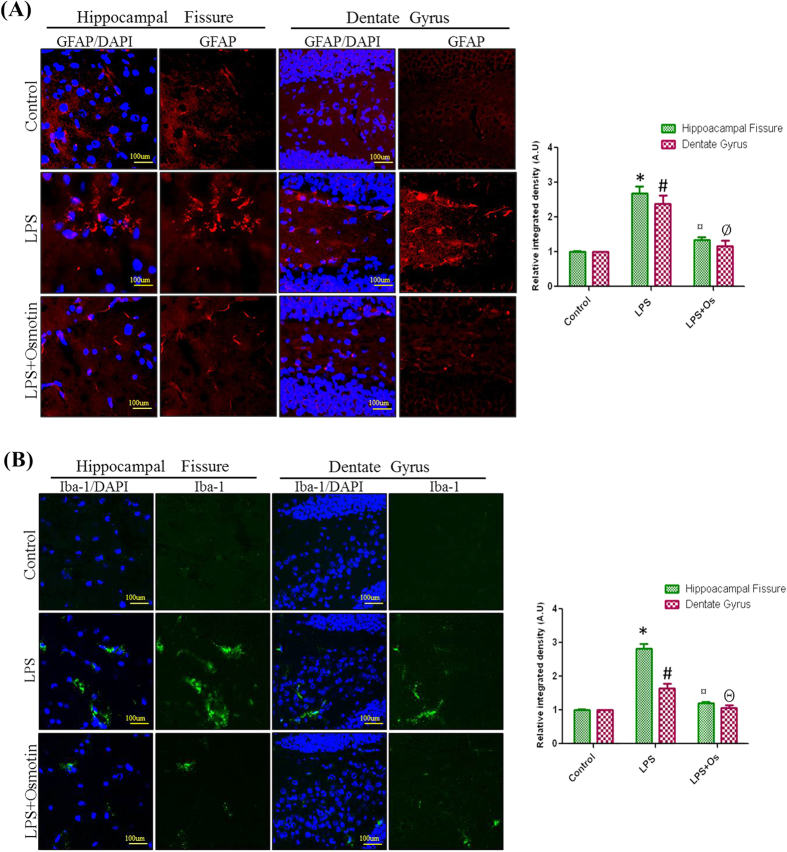
Effect of osmotin on LPS-induced activation of astrocytes and microglia in the hippocampus of adult mice. Shown are representative photomicrographs of immunofluorescence analyses of (**A**) astrocytes (GFAP-positive cells) and (**B**) microglia (Iba-1-positive cells) in the experimental groups. Images are representative of staining obtained in sections prepared from at least 5 animals per group. Panels representing the hippocampal fissure and DG region of the hippocampus show GFAP and Iba-1 stained brain tissue, respectively, at a magnification of 10x objective field, scale bar = 100 μm. Symbols for the treatment groups and levels of significance are mentioned in the data analysis section of the Materials and Methods.

The activation of microglia and astrocytes are the major elements for the progression of neuroinflammation, and activated glial cells are pools for the released inflammatory cytokines^28^,^29^. LPS treatment activates microglia and astrocytes *in vitro* or *in vivo*^2^,^30^. Therefore, we investigated the inhibitory effect of osmotin on LPS-induced astrocytes and microglial activation. Confocal microscopy revealed that systemic LPS administration significantly increased the number of activated astrocytes (GFAP-reactive cells) and microglia (Iba-1-reactive cells) in the cortex and hippocampus of adult mice. Osmotin treatment attenuated the expression of GFAP- and Iba-1-reactive cells compared to the LPS-treated group (Fig. 4).

### Osmotin inhibits LPS-induced protein expression of Aβ, APP, BACE-1, and p-Tau

**Figure 5:**
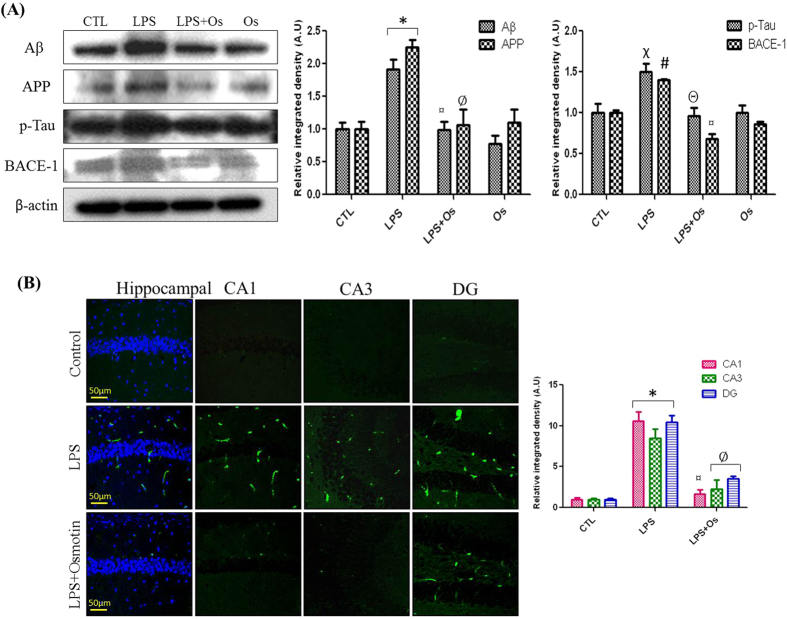
Osmotin inhibits LPS-induced protein expression of Aβ, APP, BACE-1, and p-Tau. (**A**) Shown are representative Western blots probed with Aβ, APP, BACE-1, and p-Tau antibodies in the hippocampus of adult mice. The density values are expressed in arbitrary units as the mean ± SEM for the indicated proteins (n = 5 animals per group). (**B**) Shown are representative photomicrographs of immunofluorescence analyses of Aβ-positive cells in the experimental groups. Images are representative of staining obtained in sections prepared from at least 5 animals per group. All of the panels representing the CA1, CA3, and DG regions of the hippocampus show Aβ-stained brain tissue at a magnification of 10x objective field, scale bar = 50 μm. Symbols for treatment groups and levels of significance are mentioned in the data analysis section of the Materials and Methods.

We examined the effect of LPS and osmotin on the protein expression levels of AD markers, such as Aβ, APP, p-Tau, and BACE-1, in the hippocampus of adult mice. Several studies confirmed that LPS treatment for 1 week in adult rats and mice induced AD-like effects, disrupted synaptic function and impaired memory performance^4^,^16^. Western blots demonstrated that systemic LPS administration significantly increased Aβ and APP levels compared to the control group, and osmotin treatment significantly reversed LPS-induced increases in Aβ and APP levels (Fig. 5A). Our morphological results also demonstrated the anti-AD effect of osmotin. Decreased Aβ levels were observed in the LPS plus osmotin-treated group compared to the LPS-treated group (Fig. 5B). Tau hyperphosphorylation and BACE-1 activation are also critical factors in AD progression. Therefore, we investigated the expression levels of Tau and BACE-1 in LPS- and osmotin-treated mice. Western blot analysis revealed that systemic LPS administration increased the protein expression of p-Tau and BACE-1 enzyme compared to the control group, and osmotin treatment attenuated LPS-induced Tau hyperphosphorylation and BACE-1 enzyme levels (Fig. 5A). These results were also demonstrated in cortical tissue, which also exhibited a significant reduction in Aβ and p-Tau protein expression following osmotin treatment (Fig. S3).

### Effect of osmotin on LPS-induced synaptic dysfunction and memory impairment

**Figure 6:**
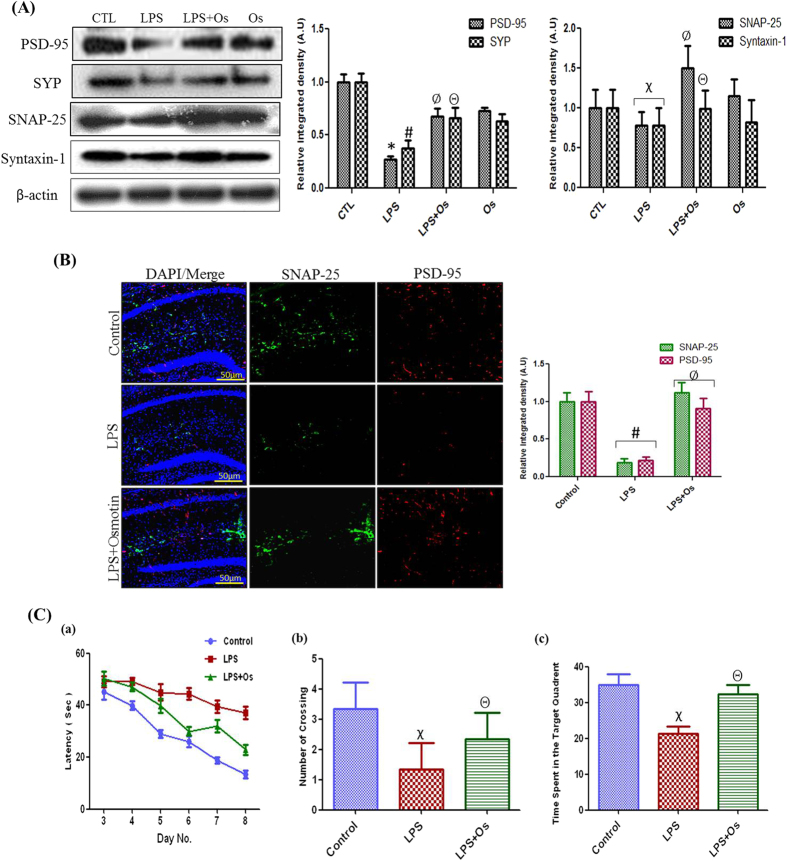
Effect of osmotin on LPS-induced synaptic dysfunction and memory impairment. (**A**) Shown are representative Western blots probed with PSD-95, SNAP-25, SYP, and syntaxin-1 antibodies in the hippocampus of adult mice. The density values are expressed in arbitrary units as the mean ± SEM for the indicated proteins (n = 5 animals per group). (**B**) Shown are representative photomicrographs of immunofluorescence analysis of PSD-95- and SNAP-25-positive cells in the experimental groups. Images are representative of staining obtained in sections prepared from at least 5 animals per group. All of the panels representing the hippocampus show PSD-95- and SNAP-25-stained brain tissue at a magnification of 10x objective field, scale bar = 50 μm. Symbols for treatment groups and level of significance are mentioned in the data analysis section of the Materials and Methods. (**C**) Behavioral studies show osmotin improves memory impairment in LPS-treated mice (n = 5 per group). (a) Average escape latency time for experimental mice to reach the hidden platform from day 3 to day 8. (b) The number of crossings at the hidden platform during the probe test of the Morris water maze experiment. (c) Time spent in the platform quadrant, where the hidden platform was placed during the trial session. Symbols for treatment groups and levels of significance are mentioned in the data analysis section of the Materials and Methods.

Recent studies demonstrated that disruption of synaptic function is a primary feature of AD and causes cognitive dysfunction and memory impairment with or without the induction of neurodegeneration^31^. Therefore, we investigated the effect of LPS and osmotin on postsynaptic (PSD-95) and presynaptic proteins, such as synaptophysin (SYP), synaptosomal associated protein (SNAP-25), and syntaxin-1. Western blot analyses revealed that systemic LPS administration decreased protein levels of PSD-95, SYP, SNAP-25, and syntaxin-1 in the hippocampus of adult mice compared to the control group. Osmotin treatment reversed the LPS-induced effect on synaptic markers and increased the expression of PSD-95, SYP, SNAP-25, and syntaxin-1 compared to the LPS-treated group (Fig. 6A). Immunofluorescence analyses also demonstrated the protective effect of osmotin on LPS-induced synaptic dysfunction. Confocal microscopy revealed a decrease in the fluorescence signals of SNAP-25 and PSD-95 in the hippocampus of the LPS-treated group compared to the control group, and LPS plus osmotin-treated mice exhibited an increased expression of SNAP-25 and PSD-95 compared to the LPS-treated group (Fig. 6B).

Mice were treated with vehicle, LPS, and osmotin, as described in the drug treatment schedule, to evaluate the memory-improving effect of osmotin (Fig. S4). All animal groups were trained to find the hidden platform in a water pool for 4 days, and the Morris water maze test was performed for 6 consecutive days. Our results demonstrated that LPS-treated mice required more time to find the hidden platform and performed fewer platform crossings compared to the control group. The osmotin-treated group required less time to find the hidden platform and performed increased platform crossings compared to the LPS-treated group. The vehicle-treated group exhibited shorter average escape latencies of 46 sec (day first) to 14 sec (day six), and the LPS-treated group exhibited average escape latencies of 49 sec (day first) to 37 sec (day six). These results demonstrate memory impairment in the LPS-treated group. Osmotin treatment significantly ameliorated the LPS-induced cognitive dysfunction, and these mice exhibited shorter average escape latencies of 50 sec (day first) to 23 sec (day six) (Fig. 6C).

A probe trial was performed 24 hrs after the water maze test in all experimental groups. The hidden platform was removed during this trial, and the average time spent in the target quadrant was measured. LPS-injected mice swam across the entire pool and spent less time in the target quadrant than the control group. Osmotin plus LPS treatment retained the LPS-induced memory impairment because these mice exhibited a significant increase in the average time spent in the target quadrant (Fig. 6C).

### Osmotin attenuates LPS-induced apoptotic neurodegeneration in the hippocampus of adult mice

**Figure 7:**
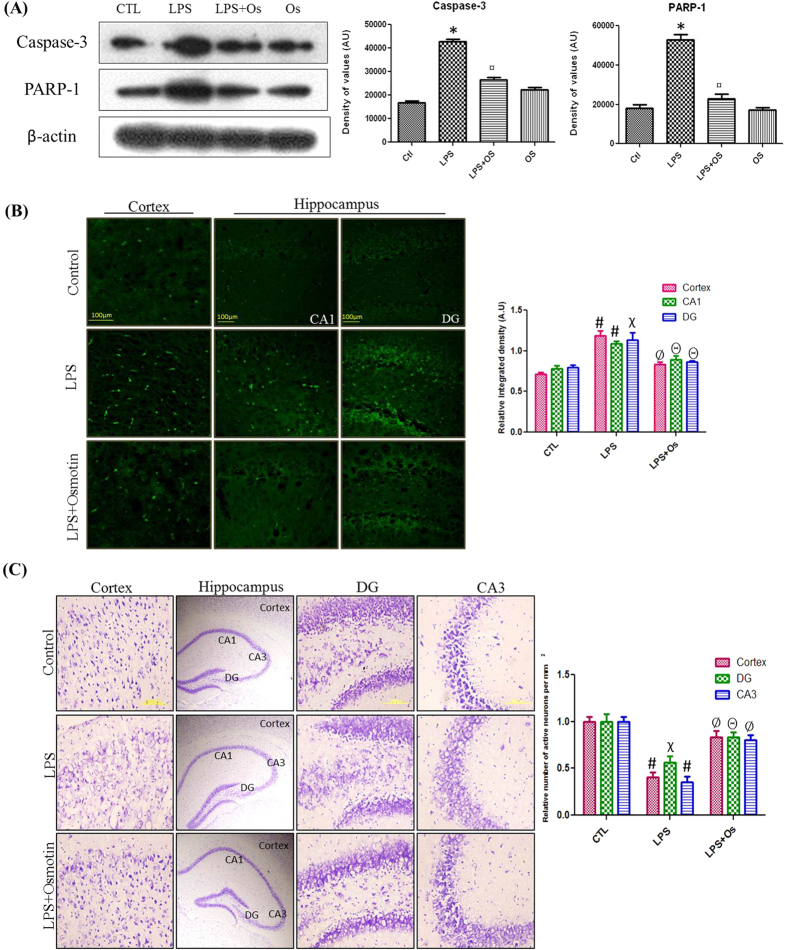
Osmotin attenuated LPS-induced apoptotic neurodegeneration in the hippocampus of adult mice (**A**) Shown are representative Western blots probed with caspase-3 and PARP-1 antibodies in the hippocampus of adult mice. The density values are expressed in arbitrary units as the mean ± SEM for the indicated proteins (n = 5 animals per group). Shown are representative photomicrographs of (**B**) FJB staining (magnification 40× objective field, scale bar = 100 μm) and (**C**) Nissl staining (magnification 20× objective field, scale bar = 200 μm) for dead and damaged neurons. Images are representative of staining obtained in sections prepared from at least 5 animals per group. Symbols for treatment groups and level of significance are mentioned in the data analysis section of the Materials and Methods.

Autocrine secretion of TNF-α and the increased production of NO are the predominant factors of LPS-induced apoptosis^32^. We investigated caspase-3 and PARP-1 protein expression to evaluate the effect of osmotin on LPS-induced apoptotic neurodegeneration. Caspase-3 is the primary executioner of apoptosis^33^, and PARP-1 overexpression induces DNA damage that is involved in the apoptotic pathway^34^. Our results demonstrated that osmotin administration significantly reduced LPS-induced elevated levels of caspase-3 and PARP-1, which supports its potential neuroprotective capability (Fig. 7A).

The neuroprotective effect of osmotin was investigated morphologically using FJB and Nissl staining in the cortex and hippocampus of adult mice. FJB is a well-known marker for degenerating neurons, and it was used to observe the level of apoptotic neurodegeneration in LPS- and osmotin-treated animals. Our results revealed that LPS treatment significantly elevated the number of degenerated neuronal cells, and osmotin treatment significantly reduced LPS-induced neurodegeneration, which was indicated by the decreased number of FJB-positive cells compared to the LPS-treated group (Fig. 7B). Nissl staining also revealed increased neuronal viability in the osmotin-treated animals compared to LPS-treated animals. Histology of the cortex and hippocampus revealed that LPS treatment increased the level of damaged, fragmented or dead neurons compared to the control group. Osmotin plus LPS administration significantly reduced the number of degenerated neurons in the cortex and hippocampus of adult mice (Fig. 7C).

## Discussion

The pathogenesis of LPS-induced neuroinflammation and its associated diseases suggest that our therapeutic drug should effectively inhibit the inflammatory processes involving neuronal and microglial cells. Activated microglia acquire macrophage-like functions during neuroinflammation that include phagocytosis and cytokine production^35^. We previously reported that osmotin may ameliorate ethanol- and Aβ-induced neurodegeneration and synaptic dysfunction in rodent brain^21^,^22^. The present study evaluated the neuroprotective potential of osmotin against neuroinflammation and its associated amyloidogenesis and apoptotic neurodegeneration in an LPS mouse model. We hypothesized that osmotin would inhibit TLR4 downstream signaling via interaction with the TLR4-CD14 receptor complex and prevent neuroinflammation-induced neurodegeneration (Fig. S5).

Activation of TLR4 and CD14 receptors is the primary event in the induction of inflammatory processes. Pathogens, especially bacterial endotoxins, specifically recognize the TLR4 substrate, and it binds to TLR4 and CD14 receptors and triggers immune responses^23^,^25^. Activation of TLR4 signaling pathways induces NFκB activation and triggers the release of inflammatory mediators, including COX-2, TNF-α, IL-1, IL-6 and iNOS^2^,^36^. LPS binds to TLR4 and CD14 receptors at the primary level and induces NFκB signaling pathway and subsequent cellular events^37^,^38^. TLR4 activation induces proinflammatory cytokines via the signaling of different adaptor proteins, such as TIRAP and MyD88, and activates NFκB and activator protein (AP)-1, which triggers the activation of other inflammatory genes^36^,^39^. Notably, recent studies demonstrated that adiponectin interfered with the TLR4-CD14 complex in cardiomyocytes via binding to AdipoR1 and prevented downstream signaling to inhibit inflammatory processes^18^. Adiponectin exhibits a promising role in inflammation-associated diseases, such as obesity, diabetes, cardiac injuries and neurological disorders^40^,^41^. Osmotin is a homologue of adiponectin, and we hypothesized that osmotin would prevent LPS-induced inflammatory processes in a similar manner as adiponectin. The established mechanisms of the beneficial effects of adiponectin or osmotin mostly describe AMPK and its related molecules. However, our study focused on the anti-inflammatory effect of osmotin via interaction with the TLR4-CD14 receptor complex. Previous studies demonstrated that the TLR4 complex contains several receptors, such as MD-2, CD14, TRIF, and MyD88, that are involved in signal processing^39^. Our results demonstrated the presence of AdipoR1 at the TLR4 site, and osmotin interacted with AdipoR1 to prevent the downstream inflammatory signals.

Chronic neuroinflammation is a pathological event that occurs during the progression of AD. Activation of neuronal microglia and reactive astrocytes during neuroinflammation are important components in AD progression^42^,^43^. The surrounding of β-amyloid deposits in AD brains may contain microlocalized acute phase reactant proteins, cytokines, and other inflammatory mediators^12^. Several studies confirmed impairments of cognitive function and increased levels of AD markers, such as Aβ, BACE-1, and p-Tau, following LPS administration in rodents^4^,^16^. Deng *et al.*^15^ recently demonstrated that LPS-induced chronic neuroinflammation developed an amyloidogenic axonal and dendritic pathology with up-regulated BACE-1 and Aβ levels in adult rat brains^15^. Our data also revealed that systemic LPS administration induced a TLR4/NFκB-based neuroinflammation that increased the levels of Aβ, p-Tau, and BACE-1 to the detriment of synaptic function. In contrast, several research studies reported an association of the TLR4/CD14 complex with innate immunity receptors on microglia that are involved in the clearance of Aβ deposits as a natural defense mechanism^44^,^45^. Recent research elucidated the mechanisms of the neuroprotective and anti-AD effects of adiponectin, which include protection against Aβ-induced oxidative stress, the insulin-sensitizing action of adiponectin, the modulation of brain metabolism and its associated cognitive impairment, and inflammation-associated memory functions^46^,^47^. The homolog of adiponectin, osmotin, also exhibited an anti-AD effect via a reduction in the protein expression levels of Aβ, APP, BACE-1, and p-Tau in adult mouse hippocampus (Fig. 6). We also demonstrated that osmotin regained synaptic functionality via elevation of the expression of pre- and post-synaptic proteins, such as SYP, SNAP-25, and PSD-95.

LPS-induced neuroinflammation activates the mitochondrial apoptotic pathway and enhances apoptotic neurodegeneration^48^. LPS-induced apoptosis was also widely investigated in other tissues, such as myocardial cells, endothelial cells, and hepatocytes^49^,^50^,^51^. *In vitro* and *in vivo* studies demonstrated that LPS induced neuronal cell death via a TLR4-dependent pathway^30^. TLR4 signaling leads to the activation of AP-1, and AP-1 promotes the JNK signaling cascade to induce neuroinflammation and neurodegeneration and/or interferes with the BCL-2 family of proteins to activate the mitochondrial apoptotic pathway^52^,^53^. Our previous reports demonstrated the neuroprotective and anti-apoptotic effects of osmotin in developmental models^21^,^22^. The present study investigated the potential anti-apoptotic behavior of osmotin in the hippocampus of adult mice. Our results demonstrated that osmotin inhibited LPS-induced DNA damage and activated the caspase cascade (Fig. 7). Overall, this study demonstrated the protective effect of osmotin against LPS-induced neuroinflammation and neurodegeneration *in vivo* and *in vitro*.

## Conclusion

The findings of the current study support the use of osmotin as a novel therapeutic agent for the treatment of neuroinflammation, neurodegeneration, and associated diseases. This study provides a new strategy to target adiponectin receptors for the treatment of neurodegenerative disorders. Our results demonstrated that the binding of osmotin to AdipoR1 at the TLR4 site inhibited LPS-induced neuroinflammatory and neurodegenerative signals. However, further research is required to evaluate the mechanistic role of osmotin in various neurodegenerative disorders.

## Materials and Methods

### *In vitro* cell culture and drug treatment

BV2 microglial cells were kindly provided by Dr. I. W. Choi (Inje University, Busan, Korea). BV2 cells were maintained in a solution of DMEM, 10% fetal bovine serum (FBS), and antibiotics (penicillin and streptomycin). BV2 cells were grown for 4 days and treated as follows: (1) Control: incubated in DMEM solution for 24 hr; (2) LPS-treatment: incubated in DMEM solution containing LPS (1 μg/ml) for 24 hr; (3) LPS + osmotin co-treatment: incubated in DMEM solution containing LPS (1 μg/ml) and osmotin (0.4 μM) for 24 hr; (4) osmotin treatment: incubated in DMEM solution containing osmotin (0.4 μM) for 24 hr; (5) TAK242 and LPS treatment: incubated in DMEM solution containing TAK242 (1 μM) for 2 hr and post-incubated with LPS (1 μg/ml) for 24 hrs; and (6) AdipoR1siRNA treatment: incubated in DMEM solution containing AdipoR1siRNA (30 pM) for 48 hr and post-incubated with LPS (1 μg/ml) and osmotin (0.4 μM) for 24 hr. All groups were treated on day 4, and cells were harvested on day 5 for use in the desired analyses.

### ApoTox-Glo, triplex assay

The ApoTox-Glo triplex assay (Promega, Promega BioSciences, LLC, San Luis Obispo, CA, USA) was performed according to our previously defined procedure^22^. Briefly, the BV2 microglial cell lines were seeded in 96-well plates (1 × 10^5^ cells/well) in 200 μl of DMEM media. Cells received respective drug treatments, and viability/cytotoxicity reagents containing a GF-AFC substrate and bis-AAF-R110 substrate were added to all wells. Cells were agitated for 30 sec and incubated for 1 hr at 37 °C. Fluorescence spectra were measured at 400/505 nm for viability assays and 485/520 nm for cytotoxicity assays. Caspase3/7 activity was assessed by the addition of caspase-Glo 3/7 reagent (100 μl) to all wells, and luminescence was measured at 485/520 nm to determine caspase activation. The results were calculated as % cell viability, % cytotoxicity, and % caspase-3/7 activity.

### Western blot analysis

Western blot analyses for cell lines were performed to determine the protein expression levels of inflammatory proteins. Proteins samples from microglial cells were collected after centrifugation and separated on Novex 4–12% Bis-Tris Plus gels (Life Technologies) under reducing conditions. Separated proteins were transferred to a polyvinylidene difluoride (PVDF) membrane. A broad range of protein markers (7–200 kDa; GangNam-STAIN, iNtRON Biotechnology) was run in parallel to detect the molecular weights of the proteins. Skimmed milk was used for membrane blocking to reduce non-specific binding. Immunoblottings were performed using respective primary antibodies and secondary antibodies. An anti-actin antibody served as the loading control. Immunocomplexes were visualized using Ez West Lumi western blotting detection reagent (Atto Corporation Tokyo). The X-ray films were scanned, and optical densities of the bands were measured using computer-based Sigma Gel software (Jandel Scientific, San Rafeal, Chicago, IL, USA).

### Immunoprecipitation

Immunoprecipitation analyses were performed to determine interactions between TLR4, CD14, and AdipoR1 receptors. Briefly, microglial cells were collected using RIPA buffer, incubated at 4 °C for 10 min, disrupted and centrifuged at 10000 × *g* to pellet cellular debris. Protein A/G plus-agarose beads (20 μl) were added to the protein sample and incubated for 2 hrs. The beads were retrieved using centrifugation at 2500 rpm. The supernatants were removed, and 10 μl of primary antibody was added (anti-TLR4, goat polyclonal, Santa Cruz Biotechnology). Samples were incubated at 4 °C overnight on a rotating device. Protein A/G agarose beads (40 μl) were added and incubated for 4 hrs. Antibody-bound beads were collected using centrifugation (2500 rpm, 5 min) and washed three times with PBS following repeated centrifugation. The final pellet was solubilized in 30 μl of 2× electrophoresis sample buffer, and the solution was boiled for 3–4 min. Proteins were analyzed using Western blot analysis.

### *In vivo* drug treatment

C657LB male mice (n = 40, Gyeongsang National University Animal Breeding Center, Jinju, South Korea) weighing 25–30 g at the start of the treatment were housed in a temperature-controlled environment and maintained on a 12-hr light/dark cycle (lights on at 6:00 am) with food available *ad libitum*. The mice were randomly divided into 4 groups as described below.

1. Control group (i.p. saline for 1 week).
2. LPS group (i.p. 250 μg/kg for 1 week).
3. LPS + osmotin (i.p. 15 μg/g, 3 times weekly).
4. Osmotin (i.p. 15 μg/g, 3 times weekly).

Figure S4 describes the drug treatment schedule. Experimental procedures were performed in accordance with the rules established by the animal ethics committee (IACUC) of the Division of Applied Life Sciences, Department of Biology, Gyeongsang National University South Korea. All of the *in vivo* experimental techniques were approved (Approval ID: 125) by the animal ethics committee (IACUC) of the Division of Applied Life Sciences, Department of Biology at Gyeongsang National University, South Korea.

### Behavioral studies (Morris water maze test)

A spatial memory test was performed for behavioral studies of experimental mice. The water maze apparatus is a circular water tank (diameter: 100 cm and height: 40 cm) filled with white ink-mixed water to a depth of 15.5 cm and maintained at 22–25 °C. The circular pool was divided into four equal quadrants, and a transparent escape platform (diameter: 10 cm and height 14.5 cm) was placed at midpoint of one quadrant. The swimming behavior of the mice was monitored and analyzed using a video tracking system (SMART, Panlab Harward Apparatus, Bioscience Company, USA). The mice were trained for 4 consecutive days before treatment and rested for 3 days. The latency to escape was calculated for 6 consecutive days. The final escape latency and probe tests were performed on the 6^th^ day to evaluate consolidated spatial memory. The platform was removed during the probe trial, and each mouse was allowed to search for the platform in the pool for 60 sec. The time spent in the target quadrant was used to evaluate the degree of memory consolidation.

### Western blot analysis

Mice were anesthetized after treatment and decapitated, and the brain samples were differentiated. Cortices and hippocampi were removed and frozen in dry ice. All brain tissues were homogenized in pro-prep extraction solution (iNtRON Biotechnology). Proteins were processed for immunoblotting as described above.

### Tissue sample preparation

Mouse brain samples (n = 5 per group) were collected after transcardial perfusion with normal saline solution (0.9%) and 4% ice-cold paraformaldehyde. Brain tissues were fixed in paraformaldehyde for 3 days followed by immersion in a 20% sucrose phosphate buffer for 3 days. Brain tissues were frozen in O.C.T. compound (A.O. Co., USA), and 12–14-μm sections were created in coronal planes (Leica cryostat CM 3050, Germany). Sections were thaw-mounted on probe-on plus-charged slides (Fisher, USA) at room temperature and stored at −70 °C.

### Immunofluorescence assays

Tissue slides were dried overnight and washed twice with 0.01 M PBS for 5 min. Tissue samples were incubated with proteinase K for 5 min, rinsed with PBS and blocked with normal serum (Vector Laboratories, 1:20 in PBS) for 60 min. Primary antibodies (TLR4, NFκB, Tnfα, IL-1β, Aβ, p-Tau, PSD95, and Snap-25) were diluted 1:100 in PBS containing 2% serum and 0.1% Triton X-100 and incubated at 4 °C overnight. FITC- or TRITC-labeled secondary antibodies (anti-rabbit, anti-goat, or anti-mouse) were diluted 1:50 in PBS and applied at room temperature for 90 min. Slides were washed twice with PBS for 5 min. The primary and secondary antibodies for double immunofluorescence were applied the following day. Glass cover slips were mounted on glass slides using fluorescent mounting medium (Dako 53023). Images were captured using a confocal laser scanning microscope (FV 1000MPE, Olympus, Japan).

### Fluoro Jade B staining

Fluoro-Jade B staining was performed according to the manufacturer protocol (cat# AG310, Millipore, USA). The tissue slides were air-dried overnight and immersed in a solution of 1% sodium hydroxide and 80% ethanol for 5 min. The slides were washed with 70% ethanol followed by distilled water for 2 min each. Then the slides were immersed in a solution of 0.06% potassium permanganate for 10 min, rinsed with distilled water and transferred to a solution of 0.1% acetic acid and 0.01% fluoro-jade B for 20 min. The slides were washed with distilled water and allowed to dry for 10 min. Slices were cleared in xylene for 5 min, glass cover slips were mounted on the slides and images were prepared with confocal laser scanning microscope (FV 1000, Olympus, Japan).

### Nissl staining

Tissue samples from control and experimental groups were washed twice with 0.01 M PBS for 5–10 min. Slides were stained with warmed a 0.5% cresyl violet solution for 10 min, rinsed with distilled water, and dehydrated in a series of graded ethanol (70%, 95% and 100%). Tissue slices were cleared in xylene for 5 min, and glass cover slips were mounted using mounting medium. Images were captured using a fluorescent light microscope.

### Data analysis and statistics

Western blot bands were scanned and analyzed using densitometry in the computer-based Sigma Gel System (SPSS Inc., Chicago, IL). Immunofluorescence results were evaluated using computer-based ImageJ software, and densities were calculated in arbitrary units. The data are presented as the mean ± standard error of mean (SEM). Data were analyzed using ANOVA followed by Student’s *t*-test. The symbol ‘*’ indicates a significant difference (*p* < 0.001), ‘^#^’ indicates a significant difference (*p* < 0.01), and ‘χ’ indicates a significant difference (*p* < 0.05) compared to their respective control groups. The symbol ‘¤’ indicates a significant difference (*p* < 0.001), ‘Ø’ indicates a significant difference (*p* < 0.01), and ‘Θ’ indicates a significant difference (*p* < 0.05) compared to LPS-treated groups. (Western blots and graph bars are represented as CTL = Control, LPS = LPS treated group, LPS + Os = LPS and osmotin treated group, and Os = Alone osmotin treated group).

## Additional Information

**How to cite this article**: Badshah, H. *et al.* Osmotin attenuates LPS-induced neuroinflammation and memory impairments via the TLR4/NFκB signaling pathway. *Sci. Rep.* **6**, 24493; doi: 10.1038/srep24493 (2016).

## Supplementary Material

Supplementary Information

## References

[b1] Badshah H. *et al.* Protective effect of lupeol against lipopolysaccharide-induced neuroinflammation via the p38/c-Jun N-terminal kinase pathway in the adult mouse brain J. Neuroimmune Pharmacol. 11, 48–60 (2016). 26139594 10.1007/s11481-015-9623-z

[b2] Parajuli B. *et al.* GM-CSF increase LPS-induced production of proinflammatory mediators via upregulation of TLR4 and CD14 in murine microglia. J. Neuroinflammation 9, 268 (2012). 23234315 10.1186/1742-2094-9-268 PMC3565988

[b3] Font-Nieves M. *et al.* Induction of COX-2 enzyme and down-regulation of COX-1 expression by lipopolysaccharide (LPS) control prostaglandin E2 production in astrocytes. J. Biol. Chem. 287, 6564–68 (2012). 10.1074/jbc.M111.327874 PMC3307308 22219191

[b4] Rosi S. *et al.* Memantine protects against LPS-induced neuroinflammation, restores behaviourally-induced gene expression and spatial learning in the rat. Neuroscience 142, 1303–15 (2006). 16989956 10.1016/j.neuroscience.2006.08.017

[b5] Okun E., Griffioen K. J. & Mattson M. P. Toll-like receptor signaling in neural plasticity and disease. Trends Neurosci. 34, 269–81 (2011). 21419501 10.1016/j.tins.2011.02.005 PMC3095763

[b6] Garate I. *et al.* Toll-like receptor inhibitor TAK242 decreases neuroinflammation in rat brain frontal cortex after stress. J. Neuroinflammation 11, 8 (2014). 24410883 10.1186/1742-2094-11-8 PMC3897306

[b7] Sambamurti K. *et al.* Gene structure and organization of the human beta-secretase (BACE) promoter. FASEB J. 18, 1034–6 (2004). 15059975 10.1096/fj.03-1378fje

[b8] Qin L. *et al.* Systemic LPS causes chronic neuroinflammation and progressive neurodegeneration. Glia 55, 453–62 (2007). 17203472 10.1002/glia.20467 PMC2871685

[b9] Sastre M. *et al.* Nonsteroidal anti-inflammatory drugs and peroxisome proliferatoractivated receptor-gamma agonists modulate immunostimulated processing of amyloid precursor protein through regulation of beta-secretase. J. Neurosci. 23, 9796–804 (2003). 14586007 10.1523/JNEUROSCI.23-30-09796.2003 PMC6740896

[b10] McGeer E. G. & McGeer P. L. Inflammatory processes in Alzheimer’s disease. Prog. neuropsychopharmacol. Biol. Psychiatry 27, 741–9 (2003). 12921904 10.1016/S0278-5846(03)00124-6

[b11] Pratico D. & Trojanowski J. Q. Inflammatory hypotheses: novel mechanisms of Alzheimer’s neurodegeneration and new therapeutic targets? Neurobiol. Aging 21, 441–3 (2000). 10858591 10.1016/s0197-4580(00)00141-x

[b12] Akiyama H. *et al.* Inflammation and Alzheimer’s disease. Neurobiol. Aging 21, 383–421 (2000). 10858586 10.1016/s0197-4580(00)00124-x PMC3887148

[b13] Griffin W. S. *et al.* Glial-neuronal interactions in Alzheimer’s disease: the potential role of a ‘cytokine cycle’ in disease progression. Brain Pathol. 8, 65–72 (1998). 9458167 10.1111/j.1750-3639.1998.tb00136.x PMC8098321

[b14] Sambamurti K. *et al.* Gene structure and organization of the human beta-secretase (BACE) promoter. FASEB J. 18, 1034–6 (2004). 15059975 10.1096/fj.03-1378fje

[b15] Deng X. *et al.* Lipopolysaccharide-induced neuroinflammation is associated with Alzheimer-like amyloidogenic axonal pathology and dendritic degeneration in rats. Adv. Alzheimer Dis. 3, 78–93 (2014). 25360394 10.4236/aad.2014.32009 PMC4211261

[b16] Lee Y. J. *et al.* Epigallocatechin-3-gallate prevents systemic inflammation-induced memory deficiency and amyloidogenesis via its anti-neuroinflammatory properties. J. Nutr. Biochem. 24, 296–310 (2013). 10.1016/j.jnutbio.2012.06.011 22959056

[b17] Teeling J. L. *et al.* The effect of non-steroidal anti-inflammatory agents on behavioral changes and cytokine production following systemic inflammation: Implications for a role of COX-1. Brain Behav. Immun. 24, 409–19 (2010). 19931610 10.1016/j.bbi.2009.11.006 PMC3098384

[b18] Jenke A. *et al.* Adiponectin protects against Toll-like receptor 4-mediated cardiac neuroinflammation and injury. Cardiovas. Res. 99, 422–31 (2013). 10.1093/cvr/cvt118 23674516

[b19] Narasimhan M. L. *et al.* Osmotin is a homolog of mammalian adiponectin and controls apoptosis in yeast through a homolog of a mammalian adiponectin receptor. Mol. Cell 17, 171–80 (2005). 15664187 10.1016/j.molcel.2004.11.050

[b20] Miele M., Costantini S. & Colonna G. Structural and functional similarities between osmotin from Nicotiana tabacum seeds and human adiponectin. PLos One 6, e16690 (2011). 21311758 10.1371/journal.pone.0016690 PMC3032776

[b21] Naseer M. I. *et al.* Neuroprotective effect of osmotin against ethanol-induced apoptotic neurodegeneration in the developing brain. Cell Death Dis. 5, e1150 (2014). 24675468 10.1038/cddis.2014.53 PMC3973231

[b22] Ali T. *et al.* Osmotin attenuates amyloid beta-induced memory impairment, tau phosphorylation and neurodegeneration in the mouse hippocampus. Sci. Rep. 5, 11708 (2015). 26118757 10.1038/srep11708 PMC4484370

[b23] Park B. S. & Lee J. O. Recognition of lipopolysaccharide pattern by TLR4 complexes. Exp. Mol. Med. 45, e66 (2013). 24310172 10.1038/emm.2013.97 PMC3880462

[b24] Zanoni I. *et al.* CD14 controls the LPS-induced endocytosis of Toll-like receptor 4. Cell 147, 868–80 (2011). 22078883 10.1016/j.cell.2011.09.051 PMC3217211

[b25] Gorina R. *et al.* Astrocyte TLR4 activation induces a proinflammatory environment through the interplay between My88-dependent NFκB signaling, MAPK, and Jak1/Stat1 pathways. Glia 59, 242–55 (2011). 21125645 10.1002/glia.21094

[b26] Delhase M., Hayakawa M., Chen Y. & Karin M. Positive and negative regulation of IkappaB kinase activity through IKKbeta subunit phosphorylation. Science 284, 309–13 (1999). 10195894 10.1126/science.284.5412.309

[b27] Lawrence T. The nuclear factor NF-kappa B pathway in inflammation. Cold Spring Harb. Perspect. Biol. 1, a001651 (2009). 20457564 10.1101/cshperspect.a001651 PMC2882124

[b28] Block M. L., Zecca L. & Hong J. S. Microglia-mediated neurotoxicity: uncovering the molecular mechanisms. Nat. Rev. Neurosci. 8, 57–69 (2007). 17180163 10.1038/nrn2038

[b29] Brown G. C. & Neher J. J. Inflammatory neurodegeneration and mechanisms of microglial killing of neurons. Mol. Neurobiol. 41, 242–7 (2010). 20195798 10.1007/s12035-010-8105-9

[b30] Lehnardt S. *et al.* Activation of innate imunity in the CNS triggers neurodegeneration through a Toll-like receptor 4-dependent pathway. Proc. Natl. Acad. USA 100, 8514–9 (2003). 10.1073/pnas.1432609100 PMC166260 12824464

[b31] Marcello E., Epis R., Saraceno C. & Di Luca M. Synaptic dysfunction in Alzheimer’s disease. Adv. Exp. Med. Biol. 970, 573–601 (2012). 22351073 10.1007/978-3-7091-0932-8_25

[b32] Xaus J. *et al.* LPS induces apoptosis in macrophages mostly through the autocrine production of TNF-alpha. Blood 95, 3823–31 (2000). 10845916

[b33] Rupinder S. K., Gurpreet A. K. & Manjeet S. Cell suicide and caspases. Vascul. Pharmacol. 46, 383–39 (2007). 17382599 10.1016/j.vph.2007.01.006

[b34] Koh D. W., Dawson T. M. & Dawson V. L. Poly(ADP-ribosyl)ation regulation of life and death in the nervous system. Cell. Mol. Life Sci. 62, 760–768 (2005). 15868401 10.1007/s00018-004-4508-y PMC11924476

[b35] Garden G. A. & Moller T. Microglia biology in health and disease. J.Neuroimmune Pharmacol. 1, 127–137 (2006). 18040779 10.1007/s11481-006-9015-5

[b36] Kawai T. & Akira S. The role of pattern-recognition receptors in innate immunity: update on Toll-like receptors. Nat. Immunol. 11, 373–384 (2010). 20404851 10.1038/ni.1863

[b37] Lakhani S. A. & Boque C. W. Toll-like receptor signaling in sepsis. Curr. Opin. Pediatr. 15, 278–82 (2003). 12806257 10.1097/00008480-200306000-00009

[b38] Chow J. C. *et al.* Toll-like receptor-4 mediates lipopolysaccharide-induced signal transduction. J. Biol. Chem. 274, 10689–92 (1999). 10196138 10.1074/jbc.274.16.10689

[b39] Akira S. & Takeda K. Toll-like receptor signalling. Nat. Rev. Immunol. 4, 499–511 (2004). 15229469 10.1038/nri1391

[b40] Tsao T. S., Lodish H. F. & Fruebis J. ACRP30, a new hormone controlling fat and glucose metabolism. Eur. J. Pharmacol. 440, 213–21 (2002). 12007537 10.1016/s0014-2999(02)01430-9

[b41] Kadowaki T. & Yamauchi T. Adiponectin and adiponectin receptors. Endocr. Rev. 26, 439–51 (2005). 15897298 10.1210/er.2005-0005

[b42] Hwang D. Y. *et al.* Alterations in behavior, amyloid beta-42, caspase-3, and Cox-2 in mutant PS2 transgenic mouse model of Alzheimer’s disease. FASEB J. 16, 805–13 (2002). 12039862 10.1096/fj.01-0732com

[b43] Nguyen M. D., Julien J. P. & Rivest S. Innate immunity: The missing link in neuroprotection and neurodegeneration? Nat. Rev. Neurosci. 3, 216–227 (2002). 11994753 10.1038/nrn752

[b44] Tahara K. *et al.* Role of toll-like receptor signalling in Abeta uptake and clearance. Brain 129, 3006–19 (2006). 16984903 10.1093/brain/awl249 PMC2445613

[b45] Reed-Geaghan E. G., Savage J. C., Hise A. G. & Landreth G. E. CD14 and toll-like receptors 2 and 4 are required for fibrillar A{beta}-stimulated microglial activation. J. Neurosci. 29, 11982–92 (2009). 19776284 10.1523/JNEUROSCI.3158-09.2009 PMC2778845

[b46] Chan K. H. *et al.* Adiponectin is protective against oxidative stress induced cytotoxicity in amyloid-beta neurotoxicity. PLos One 7, e52354 (2012). 23300647 10.1371/journal.pone.0052354 PMC3531475

[b47] Oh D. K., Ciaraldi T. & Henry R. R. Adiponectin in health and disease. Diabetes Obes. Metab. 9, 282–289 (2007). 17391153 10.1111/j.1463-1326.2006.00610.x

[b48] Hattori Y. *et al.* Insights into sepsis therapeutic design based on the apoptotic death pathway. J. Pharmacol. Sci. 114, 354–65 (2010). 21081836 10.1254/jphs.10r04cr

[b49] Cederbaum A. I., Yang L., Wang X. & Wu D. CYP2E1 sensitizes the liver to LPS- and Tnf α-induced toxicity via elevated oxidative and nitrosative stress and activation of ASK-1 and JNK mitogen-activated kinases. Int. J. Hepatol. 582790 (2012). 22028977 10.1155/2012/582790 PMC3199085

[b50] Munshi N. *et al.* Lipopolysaccharide-induced apoptosis of endothelial cells and its inhibition by vascular endothelial growth factor. J. Immunol. 168, 5860–6 (2002). 12023390 10.4049/jimmunol.168.11.5860

[b51] Dong M. *et al.* Chronic Akt activation attenuated lipopolysaccharide-induced cardiac dysfunction via Akt/GSK3β-dependent inhibition of apoptosis and ER stress. Biochem. Biophys. Acta 1832, 848–63 (2013). 23474308 10.1016/j.bbadis.2013.02.023 PMC3653446

[b52] Putcha G. V. *et al.* JNK-mediated BIM phosphorylation potentiates BAX-dependent apoptosis. Neuron 19, 899–914 (2003). 12818176 10.1016/s0896-6273(03)00355-6

[b53] Tournier C. *et al.* Requirement of JNK for stress-induced activation of the cytochrome c-mediated death pathway. Science 288, 870–874 (2000). 10797012 10.1126/science.288.5467.870

