# Supplementary material for: Osmotin attenuates LPS-induced neuroinflammation and memory impairments via the TLR4/NFκB signaling pathway
Source: Sci Rep. 2016 Apr 20;6:24493. doi: 10.1038/srep24493 (PMC4837357; doi:10.1038/srep24493)
Supplement: Supplementary Information [file srep24493-s1.pdf]

Osmotin attenuates LPS-induced Neuroinflammation and memory impairments via TLR4/NFκB signaling pathway

Haroon Badshah[1], Tahir Ali[1], Myeong Ok Kim[1]*

[1]Department of Biology and Applied Life Science (BK 21), College of Natural Sciences (RINS), Gyeongsang National University, Jinju, 660-701, Republic of Korea

Supplementary Data

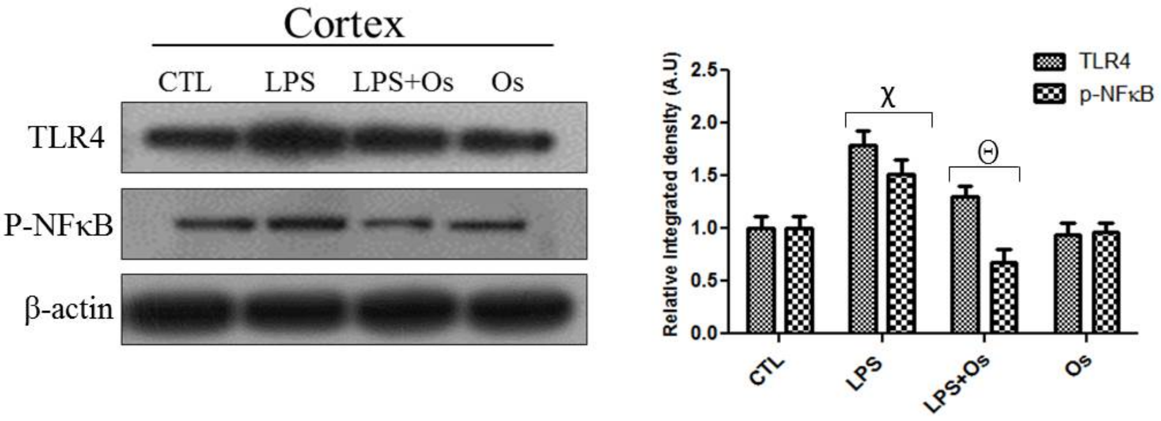

Fig. S1. Osmotin prevents LPS-induced neuroinflammatory processes via inhibiting TLR4-NfkB pathway. Shown are representative western blots probed with antibodies of TLR4 and p-NFκB in the cortex of adult mice. The protein bands were quantified using sigma gel software. The density values are expressed in arbitrary units as the mean ±SEM for the indicated proteins (n=5 animals per group). Symbols representation for treatment groups and level of significance are mentioned in data analysis section of material methods.

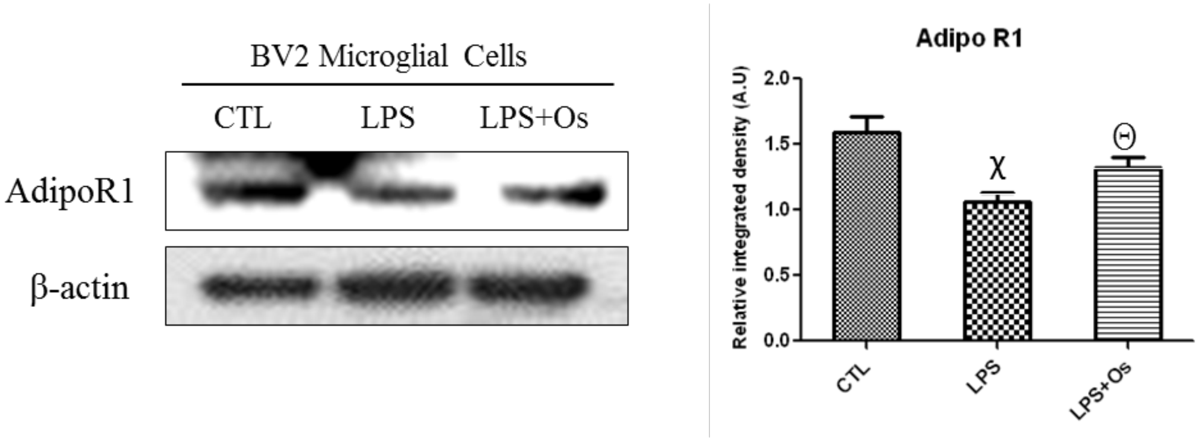

Fig. S2. Osmotin binds to AdipoR1 to inhibit neuroinflammatory signaling in BV2 microglial cells. Showed are representative immunoblots probed with antibody AdipoR1 in microglial cells. The protein bands were quantified using sigma gel software. The density values are expressed in arbitrary units as the mean ±SEM for the indicated proteins. Symbols for treatment groups and level of significance are mentioned in data analysis section of material methods.

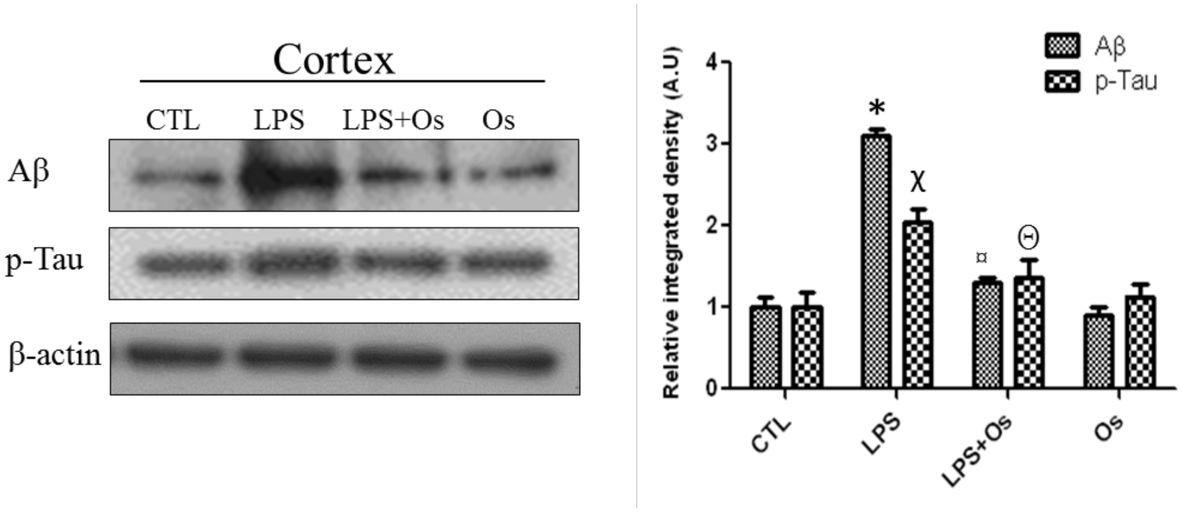

Fig. S3. Osmotin inhibits LPS-induced proteins expression of Aβ and p-Tau. Shown are representative western blots probed with antibodies of Aβ and p-Tau in the cortex of adult mice. The protein bands were quantified using sigma gel software. The density values are expressed in arbitrary units as the mean ±SEM for the indicated proteins (n=5 animals per group). Symbols for treatment groups and level of significance are mentioned in data analysis section of material methods.

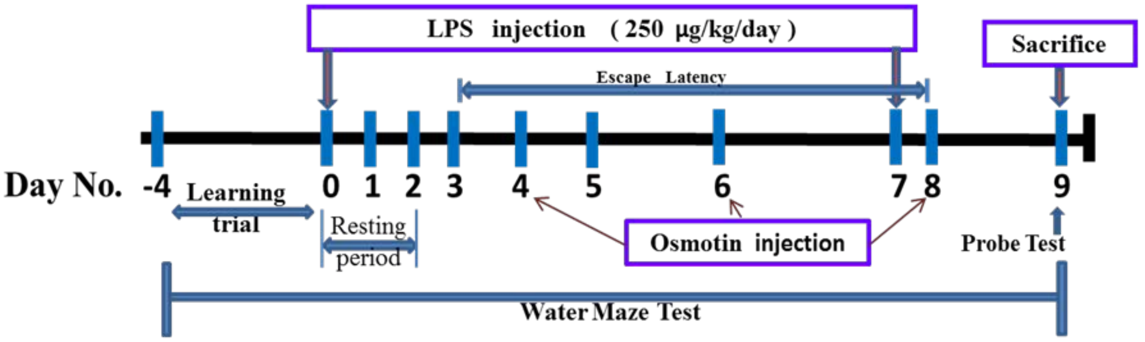

Fig. S4. Experimental scheme for effect of osmotin against LPS-induced memory impairment. Seven-week-old mice were trained for three trials per day for 4 days. Then, LPS (250 µg/kg) was administered intraperitoneally once daily for 7 days. Osmotin (15µg/g) was injected intraperitoneally along with LPS on day 4, 6 and 8. Test trials were performed subsequently.

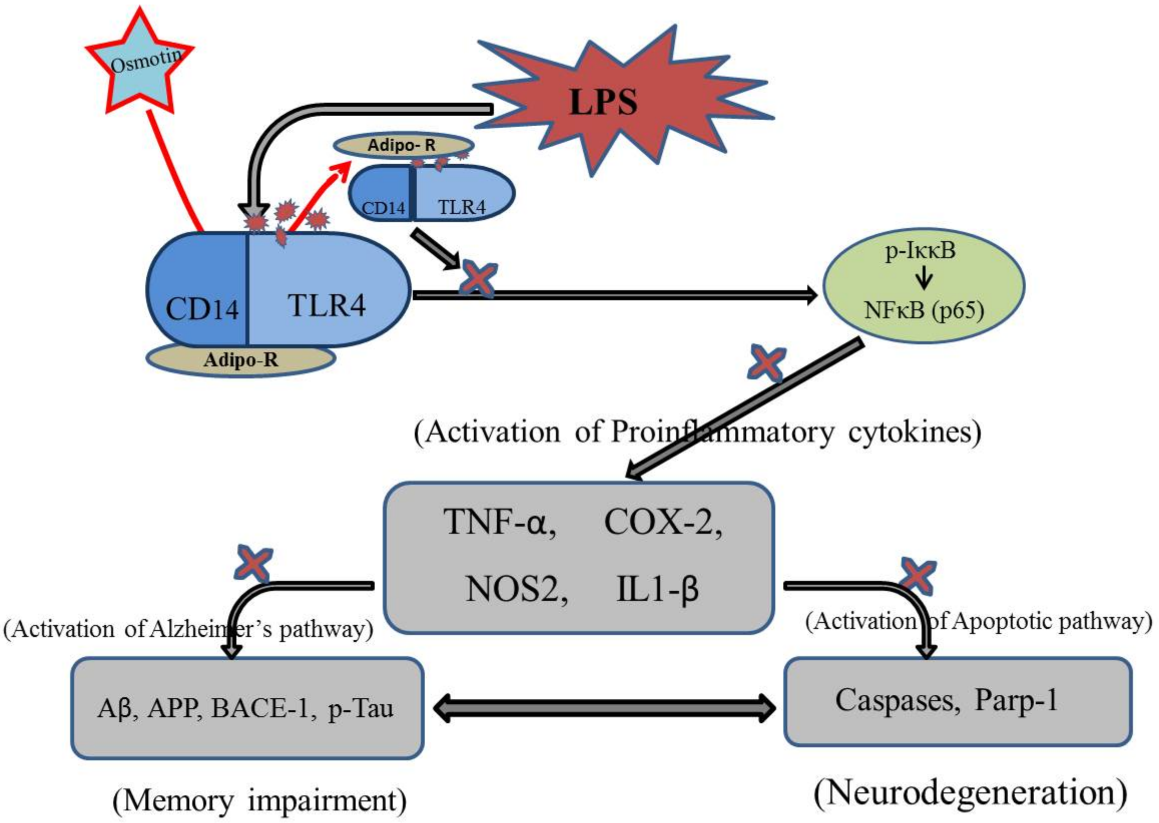

Fig. S5. Schematic diagram showing suggested neuroprotective mechanism for osmotin against LPS-induced neuroinflammation and neurodegeneration. The diagram showed possible mechanism by which osmotin prevent LPS-induced memory impairment, neuroinflammation and neurodegeneration via following the TLR4/ NFκB pathway.
